# Clinical Outcomes Following a Switch of Therapy to Faricimab in Patients Affected by Neovascular Age-Related Macular Degeneration

**DOI:** 10.3390/jcm14020423

**Published:** 2025-01-10

**Authors:** Peter Wolfrum, Elsa Wilma Böhm, Katrin Lorenz, Bernhard Stoffelns, Norbert Pfeiffer, Christina A. Korb

**Affiliations:** Department of Ophthalmology, University Medical Center of the Johannes Gutenberg-University Mainz, Langenbeckstr. 1, 55131 Mainz, Germany

**Keywords:** Faricimab, macular degeneration, AMD, anti-VEGF, Retina

## Abstract

**Objectives:** In this study, we evaluated clinical outcomes following a therapy switch to Faricimab, in a patient cohort affected by neovascular age-related macular degeneration (nAMD), having received prior intravitreal anti-VEGF therapy. **Methods:** A retrospective investigation, including 28 eyes of 23 patients, treated for nAMD at the University Medical Center Mainz, Germany was performed. A switch in therapy to Faricimab was conducted, due to an inadequate response to the previous anti-VEGF treatment. Visual acuity (VA), central retinal thickness (CRT), and axial pigment epithelial detachment (PED) height were analyzed, following the first (FU 1) and second (FU 2) Faricimab injection series. Further, a subgroup analysis was conducted to compare Faricimab responders and diminished responders, as well as an exploratory data analyses to evaluate potential influencing factors on VA and CRT changes. **Results:** The mean age of patients was 82 years, with an average prior anti-VEGF treatment duration of 4.4 years and an average of 33 prior injections. Following Faricimab, at FU 1, significant reductions in CRT (from 335.8 µm to 260.0 µm, *p* < 0.01) and axial PED height (from 177 µm to 116 µm, *p* < 0.01) were observed. At FU 2, anatomical improvements were stable. No significant improvements in VA were observed, with LogMAR remaining stable at FU 1 and FU 2. In the subgroup comparison, eight eyes fulfilled the responder criteria, exhibiting morphological and functional improvements following intravitreal Faricimab. Further, a bigger baseline CRT correlated with a bigger post-treatment CRT and a longer prior treatment duration, and a worse baseline VA correlated with a worse post-Faricimab VA. No adverse events were noted following the switch to Faricimab. **Conclusions:** Following a switch to Faricimab, significant anatomical improvements were observed, while VA remained stable. Baseline CRT, prior treatment duration, and baseline LogMAR were associated with clinical outcomes post the switch to Faricimab. Further investigations into long-term outcomes are necessary to evaluate the sustained efficacy of Faricimab.

## 1. Introduction

Age-related macular degeneration (AMD) remains the leading cause of blindness in Western countries to this day [1]. Previous epidemiological studies have shown that predominantly people over the age of 55 years are increasingly affected, and the risk of developing AMD doubles with each passing decade, reaching an annual incidence of approximately 37 per 1000 individuals for those aged 90 years and above [2,3,4]. While in 2020 around 196 million people globally were expected to be affected by AMD, due to a worldwide aging population, an increasing number of up to 290 million people are projected to be affected in 2040, according to Wong et al., which underlines the need for suitable therapeutic strategies [1].

In dry AMD, aging and increasing oxidative stress levels contribute to the apoptosis of retinal pigment epithelial cells, along with the accumulation of cellular debris, resulting in the deposition of lipofuscin and drusen [5]. Progressive degeneration eventually leads to geographic atrophy (GA) and irreversible loss of the central vision. While dry AMD typically progresses more slowly, neovascular AMD (nAMD) constitutes the majority of severe and fast progressive visual impairments and is characterized by choroidal neovascularization (CNV), secondary to the release of vascular endothelial growth factors (VEGFs).

In addition to not susceptible risk factors such as an advancing age and a genetic predisposition, smoking of tobacco constitutes the main modifiable risk factor, significantly contributing to an elevated risk of developing AMD [6].

To this day, the therapeutic options for dry AMD and geographic atrophy are very limited, whereas nAMD can be treated successfully since the beginning of intravitreal Bevacizumab injections, 20 years ago [7]. Ever since, new therapeutic options for nAMD have been developed, including anti-VEGF agents, such as Ranibizumab (Novartis; Basel), Aflibercept (Bayer; Leverkusen), or Brolucizumab (Novartis; Basel). These antibodies mainly inhibit VEGF-A, one of the central mediators of angiogenesis and have proven as effective therapy for many nAMD patients. Despite their success, a subset of patients shows suboptimal responses to existing anti-VEGF therapies, highlighting the need for novel treatment agents, also targeting alternative therapeutic pathways.

Recently, the FDA and EMA approved the first bispecific antibody, Faricimab (Roche; Basel), for the therapy of nAMD. By inhibiting VEGF-A, as well as angiopoetin 2 (Ang-2), two key pathways involved in angiogenesis are simultaneously blocked.

Ang-2, which is upregulated in pathological conditions is thereby playing a crucial role by amplifying the pro-angiogenic effects of VEGF-A and disrupting the angiopoietin 1/Tie-2 signaling pathway, which is crucial for vascular stability [8,9]. The Phase 3 clinical trials, TENAYA and LUCERNE, demonstrated promising efficacy with sustained visual function improvements lasting up to two years, along with extended intertreatment intervals, in a significant proportion of patients [8].

It has been shown many times, that outcomes in the real-world frequently are insufficient compared to the official approval trials [10,11]. Factors thereby mainly include inconsistent follow-up, as well as difference in patient characteristics and prior treatment history. This marks the importance of real-world data, to understand the true effectiveness of new therapeutic agents, as such studies provide more realistic outcomes under everyday clinical conditions.

In this study, we assess the clinical outcomes after switching to an intravitreal Faricimab therapy, in a cohort of nAMD patients, who showed insufficient response to previous anti-VEGF treatments.

## 2. Methods

### 2.1. Consent and Ethical Approval

A retrospective investigation of real-world data from the clinical routine of patients that have been treated in the Department of Ophthalmology of the Mainz University Medical Center was conducted. The publication was performed with anonymized data only, and no third party had direct access to original data. As no patients were prospectively involved in this study, ethical approval was not required, and informed consent was not obtained. This investigation complies with the regulations outlined in the ‘Landeskrankenhausgesetz, §§ 36–37’ and was conducted in compliance with the principles of the Declaration of Helsinki.

### 2.2. Selection of Patients

We considered, for inclusion, all patients that have been treated for nAMD in the Department of Ophthalmology of the Mainz University Medical Center, who had previously received an anti-VEGF therapy and were switched to intravitreal Faricimab between October of 2022 and January of 2024. Patients were treated under a pro re nata (PRN) treatment regimen and were switched to Faricimab following an inadequate response to at least one prior anti-VEGF agent. The decision of an inadequate treatment response was made by the ophthalmologist in charge of the injection consultation and considered a lack of anatomical improvement, 4 weeks after the last injection of the previous injection series. Thereby, persistent intra- or subretinal fluid and the failure to achieve significant reduction in the central retinal thickness (CRT) (<10% of baseline CRT) were determined as insufficient response. Treatment-naïve patients, missing data records, interruptions in treatment, and the presence of other comorbidities causing choroidal neovascularization or affecting the macula, such as diabetic retinopathy (DR), a history of vascular occlusion, pathological myopia, or central serous chorioretinopathy, were exclusion criteria.

### 2.3. Treatment Schedule and Injection Procedure

As mentioned prior, all patients were treated according to a pro re nata therapy scheme. Thereby, patients received an initial series of either three or four injections, which were administered every four weeks. Following the initial series, follow-up visits were conducted four weeks after the last injection. If sub- or intraretinal fluid persisted, patients received an additional injection series, including three further injections, again scheduled every four weeks.

After ocular surface anesthesia was applied, using 1% tetracaine eye drops, an eyelid speculum was inserted; the periocular surface area was then disinfected with 10% povidone–iodine and the ocular surface with 5% povidone–iodine. A thirty-gauge needle was used for the intravitreal administration of 6 mg/0.05 mL of Faricimab, through the pars plana.

### 2.4. Data Collection and Optical Coherence Tomography Imaging

The data were collected by CK and PW. The baseline assessment was defined as the date of the first Faricimab injection, with subsequent follow-up evaluations 1 (FU 1) and 2 (FU 2), four weeks following the last injection of the first and second series, respectively. We investigated epidemiological data from each patient included age, sex, laterality, prior intravitreal anti-VEGF therapy, duration since start of anti-VEGF therapy, ophthalmological comorbidities, history of prior ophthalmic surgeries, as well as safety of intravitreal Faricimab, following the switch of therapy. The decimal visual acuity (VA) was collected at baseline, as well as before every further injection during follow-up. VA was further converted to a LogMAR scale for better comparison, according to Beck et al. [12].

**Figure 1:**
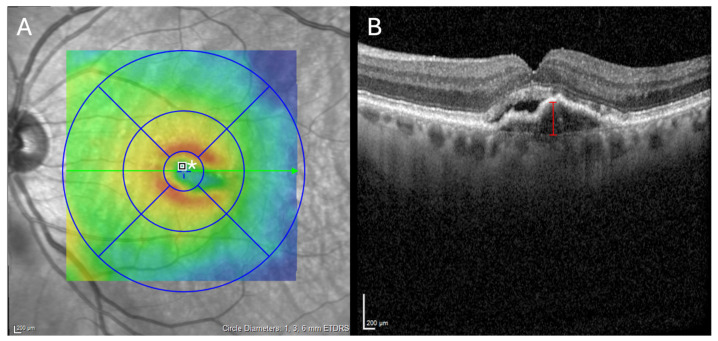
Morphological measurement. (**A**) Central retinal thickness (CRT) was measured, using the automatic Heyex 2 average thickness measurement tool within the central 1 mm^2^ area (*). (**B**) Axial pigment epithelial detachment (axial PED) height was measured manually, by quantifying the highest axial PED, reaching into the central 1 mm^2^ area (red scale).

For the analyses of anatomical changes, a 20° × 20° high-resolution Spectral-domain optical coherence tomography volume scan (Spectralis^TM^; Heidelberg Engineering; Heidelberg; Germany), which was macular-centered and included 49 b-scans, was taken at baseline and every subsequent follow-up examination. To ensure the OCT images were comparable, the automatic Heyex 2 tracking function was used. CRT measurements were conducted in the central 1 mm^2^ area using the built-in average thickness measurement tool of Heyex 2 (Heidelberg Engineering, Heidelberg, Germany). The height of the highest axial pigment epithelial detachment (PED) reaching into the central 1 mm^2^ area was manually measured, again using the measurement tools of Heyex 2 (Figure 1).

### 2.5. Outcomes

We investigated functional and morphological changes following the completion of the first and second Faricimab injection series. The VA was used as functional measure, CRT, and the height of the axial PED were applied as morphological measures. Similar to a previous Faricimab study of ours, where we analyzed differences in patient characteristics in a DME cohort, based on treatment responses after a switch of therapy to Faricimab, we again performed a subgroup comparison as part of this study, between responders and diminished responders [13]. The responder group was thereby defined as a simultaneous functional improvement of at least −0.1 LogMAR, as well as a reduction in the CRT of at least 10% compared to baseline, following the switch to Faricimab. To further investigate potential influences of age, baseline LogMAR, prior treatment duration, the number of previous anti-VEGF injections, and prior anti-VEGF agents on LogMAR and CRT, following the switch to Faricimab, an exploratory correlation analyses was conducted. Furthermore, the safety of intravitreal Faricimab was assessed.

### 2.6. Statistics

Statistical analyses were performed using SPSS, V.27 (IBM, New York, NY, USA). To compare mean values of LogMAR, CRT, and axial PED height between baseline and follow-up examinations, Wilcoxon signed-rank tests were performed. Regarding the subgroup comparison between the Faricimab responders and diminished responders, demographic data and clinical outcomes were compared between the two groups, by performing independent *t*-tests or Wilcoxon–Mann–Whitney tests, depending on the distribution of normality of the analyzed data. For the exploratory data analyses, Pearson or Spearman correlation tests were conducted, to evaluate associations between patient baseline characteristics and the follow-up LogMAR and CRT. A *p*-value of <0.05 was considered as statistically significant.

## 3. Results

The study cohort consisted of 23 patients (16 female, 7 male) with a total of twenty-eight eyes that met the inclusion criteria. All patients exhibited a macular edema at baseline, eighteen eyes (64.2%) demonstrating intraretinal fluid and nineteen eyes (67.9%) demonstrating subretinal fluid. Of the twenty-eight eyes examined, seventeen (61%) demonstrated fibrovascular PEDs, while five eyes (18%) exhibited serous PEDs. Further, drusenoid PEDs were observed in four eyes (14%), and the remaining two eyes (7%) showed no findings of a PED. The average age at the time of the initial Faricimab injection was 82 years, with patients having undergone prior anti-VEGF therapy for a mean duration of 4.4 years. During this period, patients received an average of 33 injections, as well as two different anti-VEGF agents, before the switch to Faricimab. The last injection before the switch to Faricimab was on median 35 days prior to the first Faricimab injection, and further, on average, 3.5 injections were administered in the initial Faricimab series. A summary of the complete baseline patient’s characteristics is provided in Table 1.

**Figure 2:**
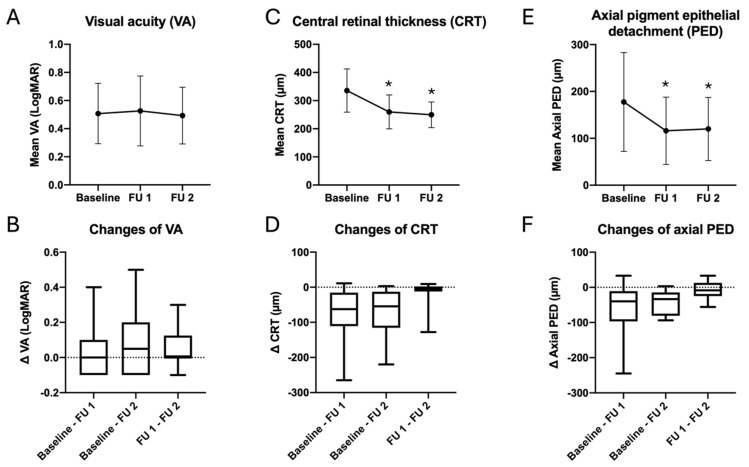
No significant improvements of the VA, following IVF (**A**). Significant reduction in the CRT (**C**) and axial PED height (**E**), following IVF. Relative changes in the VA, CRT, and axial PED height (**B**,**D**,**F**). FU 1: 3.5 ± 0.6 months after first IVF. FU 2: 7.8 ± 1.5 months after first IVF. * Statistically significant (*p* < 0.05) in comparison to baseline. Abbreviations: IVF: intravitreal Faricimab.

Following the switch to Faricimab, the LogMAR changed from 0.51 ± 0.21 at baseline to 0.53 ± 0.25 after the first injection series and to 0.49 ± 0.2 after the completion of the second series, with no statistically significant differences observed. The CRT showed a significant reduction, from 335.8 ± 77 µm at baseline to 260.0 ± 60 µm at FU 1 (*p* < 0.01), as well as to 250 ± 46 µm at FU 2 (*p* < 0.01). Similarly to the CRT, we also observed a notable decline in the height of the axial PED, from 177 ± 106 µm at baseline to 116 ± 72 µm at FU 1 (*p* < 0.01), as well as 120 ± 67 µm at FU 2 (*p* < 0.01). A summary of the complete functional and anatomical changes is provided in Figure 2 and Table 2.

In the conducted treatment–response analyses, eight eyes of the total study cohort showed a functional improvement of at least −0.1 LogMAR and a simultaneous reduction in the CRT of at least 10%, compared to baseline following a change in therapy to Faricimab. Patients of the responder group thereby had a statistically significantly bigger mean baseline CRT of 395.3 ± 73.8 μm, in comparison to eyes that did not match the responder criteria, with a mean baseline CRT of 312 ± 65.5 μm (*p* = 0.014). Regarding the further comparison between the two groups, no other statistically significant differences were observed (Table 3).

In the exploratory data analysis, a positive correlation was found between baseline CRT and post-Faricimab CRT (correlation coefficient: 0.375, *p* < 0.049) (Table 4).

Additionally, a significant and positive correlation was observed between baseline LogMAR and post-Farcimab LogMAR (correlation coefficient: 0.872, *p* < 0.001), as well as between prior anti-VEGF treatment duration and post-Faricimab LogMAR (correlation coefficient: 0.445, *p* = 0.023) (Table 5).

Following the switch to Faricimab, no adverse incidents were observed.

## 4. Discussion

We observed prompt and significant improvements in CRT and axial PED height, following the switch of therapy to Faricimab. We did not observe any statistically significant improvements in LogMAR, and the visual acuity remained stable following the switch to intravitreal Faricimab, throughout the entire observational period (Figure 2, Table 2).

By comparing the baseline characteristics of patients between the TENAYA and LUCERNE cohorts and our study cohort, we observed some distinct differences (Table 1). In our cohort, the average age at the time of the first Faricimab injection was 82 years, with a female proportion of 69% and an average prior anti-VEGF therapy duration of 53 months. In the TENAYA and LUCERNE cohorts, the average age at baseline was 76 and 75 years, respectively, with a female proportion of 57% and 61% and all patients being treatment-naïve (100%) [14]. On the other hand, the baseline CRT of 336 µm, as well as baseline LogMAR of 0.51 in our study cohort were comparable to those of the patient cohorts of TENAYA and LUCERNE (baseline CRT: 360 µm and 353 µm; baseline LogMAR: 0.48 and 0.52, conversion of ETDRS letter score according to Beck et al.) [12,14].

Similar to the anatomical improvements observed in the TENAYA and LUCERNE trials, following intravitreal Faricimab, we also noted a distinct and significant improvement of the CRT at our FU 1, following the switch to Faricimab, even though the reductions in retinal thickness were greater in the TENAYA and LUCERNE trials (−140 µm after 3.5 months) in direct comparison to our observations (−76 µm after 3.5 months) [15]. At the FU 2 examination, the anatomical improvements remained stable, consistent to findings of the TENAYA and LUCERNE cohorts [15].

Despite a similar baseline LogMAR, we did not observe functional improvements in our study cohort following intravitreal Faricimab, while in the TENAYA and LUCERNE cohorts, improvements of −0.14 LogMAR were noted at the corresponding follow-up examinations, 3.5 and 7.8 months after the switch of therapy (EDTRS letter score conversion according to Beck et al.) [12,15]. The difference in VA improvement following Faricimab might be mainly explained by the difference in patient baseline characteristics between the study cohorts. In comparison to the TENAYA and LUCERNE cohorts, the older age, lacking of treatment-naïve patients and, in particular, extensive duration of prior therapy in our cohort are well-known factors associated with a reduced potential for functional improvement [16,17]. Over the course of a prolonged anti-VEGF therapy, undertreatment as well as recurrent fluid retentions and macular edema lead to structural changes in the retina, limiting the maximum achievable visual acuity. While in total, in our study cohort the maximum achievable visual function might already have been reached, the treatment naïve cohorts of the TENAYA and LUCERNE trial had more potential to improve, even though the baseline VA before Faricimab was almost identical. A similar pattern of visual function change post-Faricimab has been observed in our prior Faricimab treatment response analysis, conducted in patients affected by DME [13].

When comparing our data with other real-world studies, similar anatomical changes in the CRT have been observed, following the switch of therapy to intravitreal Faricimab [18,19,20,21,22,23]. Further, similar anatomical improvements have also been reported concerning a distinct reduction in axial PED height, following the switch to intravitreal Faricimab [21,24,25,26].

With respect to improvements in VA, varying outcomes have been reported in numerous real-world studies, largely dependent upon the status and duration of a previous anti-VEGF therapy [19,20,21,23,27,28,29,30]. For instance, Maruyama-Inoue et al. compared the effect of intravitreal Broluzizumab to Faricimab in a cohort of only treatment-naïve nAMD patients. Thereby, following the respective anti-VEGF therapy, 4 months after the beginning of therapy, an improvement of −0.8 LogMAR was observed in the Faricimab group [19]. Furthermore, Grimaldi et al. found no VA improvement, in accordance with our results, following the switch to intravitreal Faricimab, in a similar cohort with patients’ mean age around 82 years and a comparable amount of 27 prior anti-VEGF injections [21]. On the other hand, Rush et al. conducted a retrospective analysis of a previously treated nAMD patient cohort, which received a switch to Faricimab and showed a statistically significant improvement of −0.12 LogMAR 4 months after the switch of therapy [31]. Comparing the baseline patient characteristics reveals a worse baseline VA, a lower mean age, as well as fewer prior injections and a shorter duration of therapy, compared to Grimaldi et al. and our study cohorts [21,31]. These differences thereby largely contribute to the difference in VA gain between the three compared cohorts. This observation is commonly described as the “ceiling effect” in the literature and refers to a pattern where patients with a worse VA and higher baseline CRT have a greater potential for improvement [32,33].

In our subgroup treatment–response analysis, we observed that patients categorized as part of the responder group exhibited a statistically significant bigger baseline CRT compared to those in the diminished-responder group (Table 3). When analyzing further characteristics, no other statistically significant difference between the two groups was identified. Nevertheless, a descriptive comparison revealed that patients in the responder group still displayed a worse baseline visual function compared to those in the diminished-responder group. In summary, these findings again show that patients in the responder group had an overall greater improvement potential, given worse baseline morphological and functional measures. Even though patients in the diminished responder group also showed a decline in CRT, these patients experienced limited functional improvements because they may have already reached their functional ceiling, prior to the switch to Faricimab.

The exploratory data analysis unveiled that an extended duration of prior anti-VEGF therapy was associated significantly with a worse visual function post the switch to Faricimab. Further, we also found that a worse baseline VA was linked to a worse VA following the switch to Faricimab, and a bigger baseline CRT was associated with a bigger CRT post the switch in therapy (Table 4 and Table 5). These results are thereby in line with previously reported findings in the literature, highlighting the significant role of prior treatment history and both anatomical and functional baseline status in predicting therapeutic outcomes [34].

No adverse events were recorded in our study following the intravitreal injection of Faricimab. While in November 2023, a safety warning was issued by Genentech, highlighting a potential increased risk for retinal vasculitis in patients having received intravitreal Faricimab, the year 2 reports of TENAYA and LUCERNE, published August 2024, proved the use of Faricimab as safe overall [15,35]. Most adverse events that were thereby observed in the year 2 report comprised mild and general advert ocular events, following intravitreal injections, including conjunctival hemorrhage, tears of the retinal pigment epithelium, or increased intraocular pressure. Thereby, no cases of a retinal vasculitis were observed, and only few cases of an intraocular inflammation and uveitis have been reported. The use of intravitreal Faricimab thereby proved comparable to the safety of aflibercept [15]. Still, occasional case reports of severe intraocular inflammation or retinal vasculitis have been published in the literature, which is crucial in order to be able to further analyze potential undetected health risks, as part of future meta-analyses [36,37,38,39,40,41,42]. Determining the origin of an intraocular inflammation or vasculitis in the context of a relatively new intravitreal antibody is generally challenging and also is not necessarily caused by the anti-VEGF agent itself.

The small number of our patient cohort, as well as the single-center design and retrospective approach, constitute limitations of our analysis. Further, decimal VA instead of the ETDRS letter score was measured and converted afterwards, which is generally considered less accurate for comparison to other studies. Lastly, our study focused solely on short-to-intermediate-term outcomes following the switch to Faricimab, whereas the long-term treatment course needs to be further investigated in the future to prove morphological and functional long-term stability under Faricimab.

## 5. Conclusions

Following 7 months after the switch to intravitreal Faricimab, we observed significant anatomical improvements, notably in reduction in the central retinal thickness and axial pigment epithelial height in patients affected by neovascular age-related macular degeneration. Despite these changes, we did not note functional gains in visual acuity, and LogMAR proved stable. A worse CRT at baseline was associated with a worse CRT following the switch in therapy to Faricimab, whereas a worse visual acuity at baseline as well as a longer prior treatment duration were associated with a worse visual function following the switch to Faricimab. No adverse events were recorded, following the use of intravitreal Faricimab. Further investigation into long-term clinical outcomes of patients receiving treatment for nAMD are required in the future to also assess the long-term efficiency of Faricimab.

## Figures and Tables

**Table 1:** Study cohort baseline characteristics.

Characteristics	*n* (%)
Eyes/Patients	28/23
Gender	
male	7 (30.4)
female	16 (69.6)
Age (years) at baseline examination (mean ± SD)	81.9 ± 6.3
Laterality	
right	15 (53.6)
left	13 (46.4)
Lens status	
phakia	5 (17.9)
pseudophakia	23 (82.1)
Time span (years) since first start of anti-VEGF IVI therapy (mean ± SD)	4.4 ± 3.0
Number of previous anti-VEGF treatment agents (mean ± SD)	1.9 ± 0.9
Total number of previous anti-VEGF IVI (mean ± SD)	33.3 ± 25.6
Intravitreal therapy in prior six month before Faricimab switch	
Number of anti-VEGF injections (mean ± SD)	3.6 ± 2.0
Last anti-VEGF agent, Aflibercept/ Bevacizumab/ Ranibizumab/ Brolucizumab	14/2/10/2
Number of Faricimab injections in initial series (mean ± SD)	3.5 ± 0.6

Abbreviation: VEGF: vascular endothelial growth factor; IVI: intravitreal injection.

**Table 2:** Change in VA, CRT, and axial PED.

	Baseline Examination	FU 1 Examination	FU 2 Examination
Baseline LogMAR	0.51 ± 0.21	0.53 ± 0.25	0.49 ± 0.2
Baseline CRT (µm)	335.8 ± 76.7	260.0 ± 60.1	249.6 ± 45.6
Baseline axial PED (µm)	177.7 ± 105.5	116.1 ± 71.7	120 ± 67.4

Abbreviations: VA = visual acuity; CRT = central retinal thickness; axial PED = axial pigment epithelial detachment.

**Table 3:** Univariate analysis of Faricimab treatment–response following intravitreal Faricimab.

	Responder (*n* = 8)	Diminished Responder (*n* = 20)	*p*-Value
Age (years) at first Faricimab injection (mean ± SD)	84.1 ± 6.6	81.0 ± 6.2	0.154
LogMAR at baseline (mean ± SD) FU 1 LogMAR (mean ± SD)	0.57 ± 0.2 0.47 ± 0.2	0.49 ± 0.2 0.54 ± 0.3	0.327 0.576
CRT at baseline (μm) (mean ± SD) FU 1 CRT (μm) (mean ± SD)	395.3 ± 73.8 244.9 ± 29.5	312 ± 65.5 266.0 ± 68.3	0.014 * 0.559
Axial PED height at baseline (mean ± SD) FU 1 axial PED height (mean ± SD)	172.6 ± 45.1 108.1 ± 31.7	179.8 ± 123.6 119.4 ± 83.7	0.710 0.577
Number of prior anti-VEGF injections (mean ± SD)	36.8 ± 28.3	32.0 ± 25.1	0.541
Number of prior anti-VEGF agents (mean ± SD)	2.25 ± 0.7	1.8 ± 1	0.131

Note: Responder group defined as improvement of at least −0.1 LogMAR as well as simultaneous reduction in the CRT of at least 10% of baseline CRT; VEGF: vascular endothelial growth factor; * = *p* < 0.05.

**Table 4:** Explorative data analysis of baseline variables on the CRT following Faricimab therapy switch.

	Correlation Coefficient	*p*-Value
Age	−0.247	0.205
CRT at baseline	0.375	0.049 *
Time span (years) since start of IVI therapy	−0.222	0.265
Number of previous anti-VEGF IVI	0.021	0.916
Number of previous anti-VEGF treatment agents	−0.005	0.979

Note: IVI: intravitreal injection; VEGF: vascular endothelial growth factor; * *p* < 0.05.

**Table 5:** Explorative data analysis of baseline variables on visual acuity following Faricimab therapy switch.

	Correlation Coefficient	*p*-Value
Age	−0.025	0.901
LogMAR at baseline	0.872	<0.001 *
Time span (years) since start of IVI therapy	0.445	0.023 *
Number of previous anti-VEGF IVI	0.304	0.123
Number of previous anti-VEGF treatment agents	0.157	0.435

Note: IVI: intravitreal injection; VEGF: vascular endothelial growth factor;* *p* < 0.05.

## Data Availability

The data presented in this manuscript is not publicly accessible, due to privacy restrictions.
